# Efficacy and safety of eltrombopag in Chinese patients with refractory or relapsed severe aplastic anemia

**DOI:** 10.1038/s41598-023-45607-0

**Published:** 2023-11-02

**Authors:** Hong Chang, Guangsheng He, Rong Fu, Fei Li, Bing Han, Tao Li, Lei Liu, Hemant Mittal, Hantao Jin, Fengkui Zhang

**Affiliations:** 1 West China Hospital of Sichuan University, Chengdu, China; 2 The First Affiliated Hospital of Nanjing Medical University, Jiangsu Province Hospital, Collaborative Innovation Center for Cancer Personalized Medicine, Nanjing, China; 3 Tianjin Medical University General Hospital, Tianjin, China; 4 The First Affiliated Hospital of Nanchang University, Nanchang, China; 5 Peking Union Medical College Hospital, Beijing, China; 6 Novartis Pharma Co., Ltd., Beijing, China; 7 Novartis Healthcare Private Limited, Hyderabad, India; 8 Institute of Hematology & Blood Disease Hospital, Chinese Academy of Medical Sciences & Peking Union Medical College, Tianjin, China

**Keywords:** Anaemia, Drug development

## Abstract

For patients with severe aplastic anemia (SAA) in China who have had an insufficient response to the first-line treatment with hematopoietic stem cell transplantation or immunosuppressive therapy, there is no established standard of care other than transfusion support and treatment of infections. This non-randomized, open-label, Phase II multicenter trial investigated the efficacy and safety of eltrombopag in 20 adult Chinese patients with refractory or relapsed (r/r) SAA. The primary endpoint of hematologic response rate at Week 26, defined as the proportion of patients who met any of the International Working Group criteria, was observed in 70% (14/20) of patients, with more than 50% of these having at least bi-lineage response. Reduced red blood cell and platelet transfusion at Week 26 were observed in 57% (8/14) and 80% (8/10) of patients, respectively. Safety findings were consistent with the established safety profile of eltrombopag and no new safety signals were reported. None of the patients discontinued eltrombopag because of safety concerns. Although the sample size was small, this is the first prospective study to show that eltrombopag is efficacious and has a favorable safety profile in a Chinese patient population with r/r SAA.

**Trial registration**: This trial is registered on ClinicalTrials.gov (NCT03988608); registered 17 June 2019.

## Introduction

Severe aplastic anemia (SAA) is a rare, life-threatening acquired bone marrow disorder characterized by pancytopenia and hypocellular bone marrow^1–3^. Incidence is characterized by a bimodal distribution curve, peaking among individuals between 15 and 25 years of age and also in those over 60 years of age, with equal distribution between male and female individuals. The annual incidence of SAA varies by location, with Western countries having ≈2 cases per million; however, this incidence increases up to threefold in East Asian countries^1, 4, 5^. In China, the annual incidence is recorded as 7.4 per million and follows the same bimodal distribution curve in the patient population^3^.

The China Aplastic Anemia Consensus released in 2017 by the Chinese Medical Association recommends hematopoietic stem cell transplantation (HSCT) treatment as the first-line regimen for the treatment of SAA^3^. However, this treatment option is limited in its availability to patients aged ≤ 35 years with suitable donors and carries a risk of infectious complications, graft-versus-host disease, and graft failure^6^. Up to 40% of patients without a suitable donor continue to have severe symptoms and are at risk of life-threatening complications^7^. The standard first-line therapy for patients with SAA who are not eligible for HSCT is immunosuppressive therapy (IST), such as rabbit-, horse-, or pig-derived antithymocyte globulin (ATG), administered in combination with cyclosporine A (CsA)^2, 3, 8^. SAA that does not respond to or relapses after first-line IST is known as refractory or relapsed (r/r) SAA^7^. For patients with SAA in China who are refractory to or have relapsed after first-line treatments, there is no established standard of care available, except for supportive care such as transfusion support and treatment of concurrent infections^3^. Evidence suggests that, even with support, a number of patients with r/r SAA will die within 5 years of diagnosis^9^. As such, there is a high unmet need for an effective and well-tolerated treatment regimen in these patients.

Eltrombopag is a small-molecule oral thrombopoietin receptor agonist that promotes megakaryopoiesis and increases platelet counts. It is approved for the treatment of SAA after an insufficient response to IST in the United States^10^ and in Europe^11^, and recently received approval in China. It is also approved for the treatment of persistent or chronic immune thrombocytopenia in several territories, including China, Europe, and the United States^10, 11^. There is evidence that eltrombopag can improve response rates in patients with r/r SAA^6, 7, 12^. In this study, we aimed to assess the efficacy and safety of eltrombopag in adult Chinese patients with r/r SAA.

## Methods

This is an ongoing non-randomized, open-label, single-arm, multicenter Phase II trial (NCT03988608; registered 17/06/2019) investigating the efficacy and safety of eltrombopag in Chinese patients with r/r SAA. Eligible Chinese patients were ≥ 18 years with a previous diagnosis of SAA (defined as bone marrow cellularity < 25%, or 25–50%, with ˂ 30% residual hemopoietic cells and ≥ 2 of the following in peripheral blood samples: absolute neutrophil count ˂ 0.5 × 10^9^/L, platelet count ˂20 × 10^9^/L, or absolute reticulocyte count ˂ 20 × 10^9^/L). Patients included in the study had an insufficient response after ≥ 1 IST regimen containing ATG, antilymphocyte globulin (ALG) and/or CsA, cyclophosphamide, or alemtuzumab > 6 months before study entry, and platelet count ≤ 30 × 10^9^/L at screening. Patients who did not have the option of undergoing HSCT, either because of ineligibility or non-availability of a suitable donor, were included. Exclusion criteria included patients treated with ATG/ALG, cyclophosphamide, or alemtuzumab in the past 6 months (patients who received cyclosporine or anabolic steroids [excluding danazol] at a stable dose could be enrolled if laboratory values were stable); diagnosis of congenital aplastic anemia; past medical history of thromboembolism in the 6 months before enrollment, or current use of anticoagulants; aspartate aminotransferase or alanine aminotransferase ≥ 3 × upper limit of normal (ULN); creatinine, total bilirubin, and alkaline phosphatase ≥ 1.5 × local ULN.

All patients received a starting dose of eltrombopag monotherapy of 25 mg/d. This dose increased by 25 mg/d every 2 weeks, according to the platelet count, up to the maximum dose of 150 mg/d (Supplementary Fig. S1).

The primary endpoint was the hematologic response (HR) rate at Week 26, defined as the proportion of patients who met any of the International Working Group criteria^13^ for changes in platelet count, hemoglobin level, or neutrophil count listed in Supplementary Table S1. Additional efficacy and safety assessments included HR rates at Weeks 13 and 52 (not reported here); time to HR and duration of HR; changes in platelet/hemoglobin/neutrophil count (in the absence of platelet/red blood cell transfusions/granulocyte colony-stimulating factor [G-CSF]); changes in transfusion need; safety outcomes; eltrombopag plasma pharmacokinetic parameters (including trough concentrations); and the rate of clonal evolution.

In addition to the HR reported by investigators in the response assessment case report form, HR was also derived from International Working Group criteria programmatically using laboratory results and transfusion records, and was referred to as the derived HR.

During this study, the COVID-19 pandemic resulted in the implementation of changes to the conduct of the study to ensure the safety and well-being of study patients, and to enable trial oversight and compliance with the study protocol. These protocol amendments in line with COVID-19 regulations included changes in (1) study procedures, allowing for replacement of on-site monitoring by remote monitoring at some sites when considered necessary, (2) study drug supply method, that allowed for home delivery of the study treatment, and (3) remote monitoring of study sites as applicable. These unforeseen changes were implemented as of March 2020 based on needs of individual sites to address the challenges of the pandemic. There were no reports of suspected or confirmed cases or deaths related to COVID-19 in this trial at the cutoff date.

Only data available at the cutoff date of July 16, 2021, are reported here.

### Ethics approval

This study was performed in accordance with the International Council for Harmonisation E6 Guideline for Good Clinical Practice that have their origin in the Declaration of Helsinki and local regulations (China). All patients provided written informed consent for their participation in the study. The study protocol and all amendments were approved by the Independent Ethics Committee for each center (Institute of Hematology & Blood Diseases Hospital, Tianjin, China; The First Affiliated Hospital of Nanjing Medical University, Nanjing, Jiangsu Province, China; West China Hospital of Sichuan University, Chengdu, Sichuan, China; Tianjin Medical University General Hospital, Tianjin, China; The First Affiliated Hospital of Nanchang University, Nanchang, Jiangxi Province, China).

## Results

In total, 20 patients were enrolled in the study; 85% (17/20) of patients completed the initial 26-week treatment phase while 15% (3/20) of patients discontinued the study prior to Week 26 due to patients’ decision and were considered non-responders. Patient disposition is summarized in Supplementary Fig. S2. 

Baseline demographics and baseline characteristics are presented in Table 1. All patients were Chinese. Overall, the median (range) age was 37.5 (18–69) years, and 75% (15/20) of patients were male. During the study period, the median (range) duration of exposure to eltrombopag was 258.5 (69–443) days, and the median (range) daily dose was 122.8 (26–138) mg/d. A total of 75% (15/20) of patients reached the maximal dose of eltrombopag 150 mg/d in this study.

**Table 1:** Selected demographic and baseline characteristics (full analysis set).

Parameter	Eltrombopag (N = 20)
Age, years	
Mean (SD)	39.0 (13.3)
Median (min–max)	37.5 (18–69)
Age category, n (%)	
18 to 64 years	19 (95.0)
≥ 65 years	1 (5.0)
Sex, n (%)	
Male	15 (75.0)
Race, n (%)	
Asian (Chinese)	20 (100.0)
Weight, kg	
Mean (SD)	69.7 (12.6)
Height, cm	
Mean (SD)	168.1 (7.6)
Platelet count, × 10^9^/L	
Mean (SD)	10.8 (7.9)
Hemoglobin, g/L	
Mean (SD)	66.9 (19.9)
Absolute neutrophil count, × 10^9^/L	
Mean (SD)	1.2 (1.4)
Absolute reticulocyte count, × 10^9^/L	
Mean (SD)	39.5 (33.6)^a^
RBC transfusion–independent at BL, n (%)	6 (30.0)
Platelet transfusion–independent at BL, n (%)	10 (50.0)
Concomitant CsA or anabolic steroid use at BL, n (%)	17 (85.0)^b^
Prior ATG/ALG therapies, n (%)	19 (95.0)
G-CSF injection at BL, n (%)	6 (30.0)
SAA characteristics at BL, n (%)	
Refractory	16 (80.0)
Relapsed	4 (20.0)

ALG, anti-lymphocyte globulin; ATG, anti-thymocyte globulin; BL, baseline; CsA, cyclosporin A; G-CSF, granulocyte colony-stimulating factor; RBC, red blood cell; SAA, severe aplastic anemia; SD, standard deviation.

^a^The mean of absolute reticulocyte count was calculated based on 16 patients with original value, no derivation was used.

^b^At BL, 13 patients were receiving concomitant CsA and 9 patients were receiving concomitant anabolic steroids.

**Figure 1:**
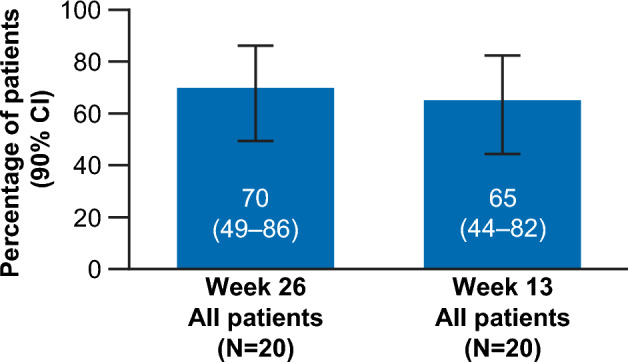
Hematologic response rate, according to IWG criteria at Week 26 (primary endpoint) and Week 13 (secondary endpoint) (full analysis set). CI, confidence interval; IWG, International Working Group.

**Figure 2:**
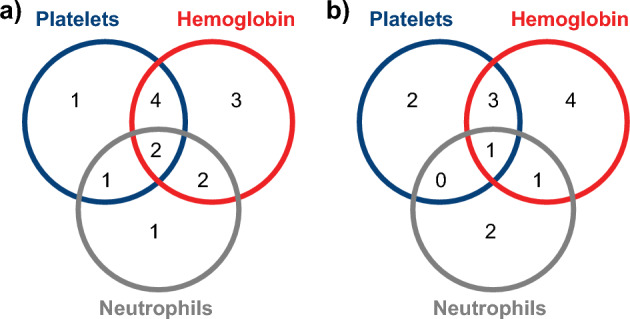
Number of responders with uni-, bi-, or tri-lineage response at Week 26 (**a**) and Week 13 (**b**) (full analysis set).

**Figure 3:**
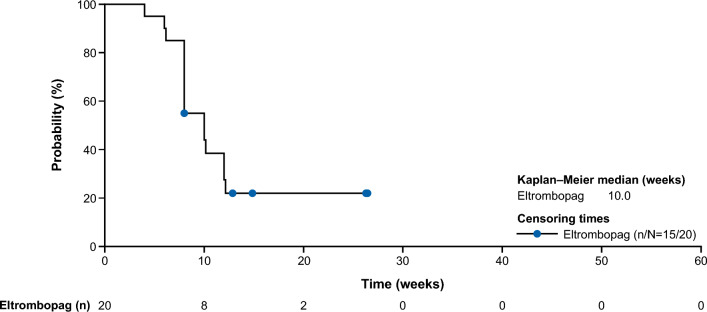
Kaplan–Meier plot of time to hematologic response by investigator (full analysis set). Event = patient achieved first hematologic response; censored = patient does not meet hematologic response prior to or on the cutoff date (July 16, 2021).

**Figure 4:**
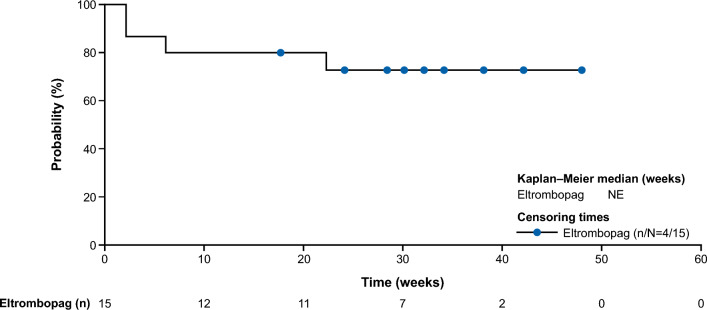
Kaplan–Meier plot of duration of hematologic response by investigator (full analysis set). Event = patient relapsed; censored = patient still responding at the cutoff date (July 16, 2021). NE, not estimable.

The primary endpoint of HR rate at Week 26, based on investigator decision, was met in 70% (14/20; 90% confidence interval [CI], 49–86) of patients (Fig. 1), with 14% (2/14) of responders having tri-lineage response, 50% (7/14) of responders having bi-lineage response, and 36% (5/14) of responders having uni-lineage response (Fig. 2A). The derived HR also showed comparable results to the primary analysis, with 70% (14/20) of patients meeting the HR at Week 26 (Supplementary Fig. S3). The secondary endpoint of HR rate at Week 13 was met in 65% (13/20; 90% CI 44–82) of patients (Fig. 1), with 8% (1/13), of these having tri-lineage response, 31% (4/13) of these having bi-lineage response, and 61% (8/13) of these having uni-lineage response (Fig. 2B). In terms of time to response, 75% (15/20) of patients achieved an HR with a median (90% CI) time of 10 (8–12) weeks per the investigator assessment using a Kaplan–Meier method (Fig. 3). Correspondingly, hemoglobin response was reported in 60% (12/20) of patients with a median of 12.1 weeks (90% CI 8.0–not estimable [NE]), platelet response in 50% (10/20) of patients and neutrophil response in 45% (9/20) of patients, both with a median time of 30 weeks (90% CI 12–NE; Table 2). For the 15 patients who had responded at any time during the first 26 weeks of the study, the median duration of HR (Fig. 4), hemoglobin response, and platelet response was not achieved by the cutoff date. Of these 15 responders, 11 patients who had the response continuing until the data cutoff date were censored. The median (90% CI) duration of neutrophil response was 6.1 (2.1–20.1) weeks. Overall, there was a trend observed toward improvement in hematologic values (platelet count, hemoglobin level, and neutrophil count) up to Week 26. Median interquartile range (IQR) platelet count at baseline was 9.3 × 10^9^/L (5.3–14.5) and increased to 18.0 × 10^9^/L (6.0–29.0) at Week 13, and 30.0 × 10^9^/L (7.0–77.0) at Week 26 (Supplementary Fig. S4A). Median (IQR) hemoglobin count at baseline was 65.5 g/L (52.0–76.0) and increased to 82.0 g/L (63.0–118.0) at Week 13, and 90.0 g/L (81.0–127.0) at Week 26 (Supplementary Fig. S4B). Neutrophil count changed from a baseline median (IQR) of 0.8 × 10^9^/L (0.5–1.1) to 1.2 × 10^9^/L (0.8–1.4) at Week 13, and 1.2 × 10^9^/L (0.9–1.7) at Week 26 (Supplementary Fig. S4C).

**Table 2:** Time to hematologic response (full analysis set).

	Eltrombopag N = 20			
	Hematologic response	Hemoglobin response	Platelet response	Neutrophil response
No. of events, n (%)	15 (75.0)	12 (60.0)	10 (50.0)	9 (45.0)
No. of censored, n (%)	5 (25.0)	8 (40.0)	10 (50.0)	11 (55.0)
KM estimates (weeks) (90% CI) at				
25th percentile	8.0 (6.0–8.0)	8.0 (6.1–12.0)	12.0 (8.0–13.9)	10.0 (6.0–18.0)
75th percentile	12.1 (10.1–NE)	NE (18.0–NE)	NE (30.0-NE)	NE
Median time to response, Weeks (90% CI)	10.0 (8.0–12.0)	12.1 (8.0–NE)	30.0 (12.1–NE)	30.0 (12.1–NE)

Event = patient achieved first hematologic response; censored = patient does not meet hematologic response prior to or on the cutoff date (July 16, 2021).

Censoring was performed using the date of the last assessment.

Percentiles with 90% CIs are calculated from PROC LIFETEST output using the method of Brookmeyer and Crowley^17^.

CI, confidence interval; KM, Kaplan–Meier, NE, not estimable.

**Figure 5:**
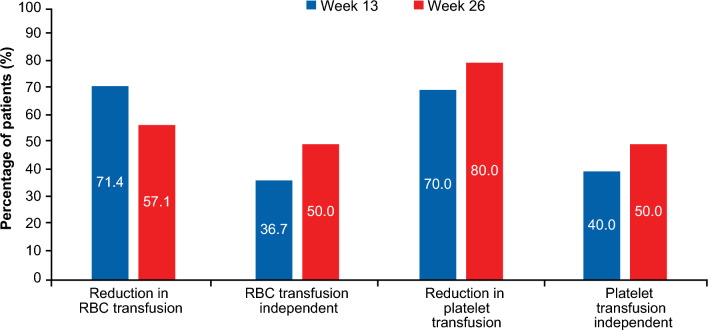
Percentage of RBC or platelet transfusion-dependent patients at baseline with a reduction or complete transfusion independence at Weeks 13 and 26**.** The denominator is the number of patients who were RBC/platelet transfusion dependent at baseline; RBC N = 14; Platelet N = 10. RBC, red blood cell.

Baseline transfusion dependency was defined as patients receiving ≥ 1 red blood cell (RBC) transfusion 8 weeks prior, or ≥ 1 platelet transfusion 4 weeks prior to study treatment. Post-baseline transfusion independence is achieved if patients who were transfusion dependent become transfusion independent for a period of ≥ 56 days (RBC) or ≥ 28 days (platelets). Of the 14 patients who were RBC transfusion-dependent at baseline, 71% (10/14) and 57% (8/14) experienced a reduction in RBC transfusions or were transfusion-independent at Weeks 13 and 26, respectively (Fig. 5). Additionally, 79% (11/14) of these patients became RBC transfusion-independent for ≥ 56 days at some point during the study. Of note, the other 6 patients who were RBC transfusion-independent at baseline remained so at the data cutoff date. The median (range) of the maximum duration of the RBC transfusion-free period was 100 days (25–337 days). Of the 10 platelet transfusion-dependent patients at baseline, 70% (7/10) and 80% (8/10) had reduced platelet transfusion or were platelet transfusion-independent at Weeks 13 and 26, respectively (Fig. 5). Moreover, all 10 patients who were platelet transfusion-dependent at baseline became platelet transfusion–independent for ≥ 28 days at some point during the study. Of note, the other 10 patients who were platelet transfusion-independent at baseline remained so until the data cutoff date. The median (range) of the maximum platelet transfusion-free period was 180.5 (33–337) days.

Adverse events (AEs) were reported in 95% (19/20) of patients, with 70% (14/20) of patients recorded as having experienced a treatment-related AE, and 15% (3/20) of patients having a grade ≥ 3 treatment-related AE (Table 3). The most common all-grade AEs were upper respiratory tract infection (30%), increase in blood creatinine levels (25%), and hyperuricemia (25%; Supplementary Table S2), and for the majority of AEs, each AE was reported in one or two patients.

**Table 3:** Overview of AEs and AEs of special interest (safety set).

AEs	Eltrombopag N = 20	
	All grades n (%)	Grade ≥ 3 n (%)
All deaths	1 (5.0)	1 (5.0)
AEs	19 (95.0)	7 (35.0)
Treatment-related	14 (70.0)	3 (15.0)
SAEs	6 (30.0)	5 (25.0)
Treatment-related	2 (10.0)	2 (10.0)
Fatal SAEs	1 (5.0)	1 (5.0)
AEs leading to dose adjustment/interruption	3 (15.0)	2 (10.0)
AEs requiring additional therapy	16 (80.0)	7 (35.0)
AEs of special interest		
Hepatotoxicity (all indications)	10 (50.0)	0
Acute kidney injury	6 (30.0)	1 (5.0)

Numbers (n) represent counts of patients.

A patient with multiple severity grades for an AE is only counted once under the maximum grade.

MedDRA version 24.0, CTCAE version 4.03.

AE, adverse event; CTCAE, Common Terminology Criteria for Adverse Events; MedDRA, Medical Dictionary for Regulatory Activities; SAE, serious adverse event.

Identified AEs of special interest (AESIs) were hepatotoxicity and acute kidney injury. Hepatotoxicity AESIs were reported in 50% (10/20) of patients; 45% (9/20) of patients experienced events that were considered treatment related. However, none of the hepatotoxicity events were grade ≥ 3. High ALT and AST levels were recorded in 2 patients by the investigator; however, these were isolated, transient increases in transaminases without evidence for severe liver injury or hepatic impairment. Hyperbilirubinemia contributed to high indirect bilirubin in 3 out of the 4 patients for whom it was recorded. There were no other liver test abnormalities or direct bilirubin increases observed in these patients; these results may be explained by interference with the laboratory for total bilirubin due to known coloration effect of eltrombopag. Acute kidney injury AESIs were reported in 30% (6/20) of patients; these events were considered treatment related in 15% (3/20) of patients. One (5%) patient had a grade ≥ 3 acute kidney injury AESI (Supplementary Table  S3). Overall, there were no AEs leading to treatment discontinuation. Of note, there were 2 cases of treatment-related severe AEs, namely osteonecrosis (n = 1) and groin pain (n = 1). Lastly, 1 death from grade 4 gastrointestinal bleeding was reported 45 days after the last dose of eltrombopag; however, this was not considered related to eltrombopag treatment. There were no on-treatment deaths reported as of the cutoff date. Clonal cytogenic evolution was detected in 1 patient at Week 13, who had a normal karyotype at baseline. This event was transient and per investigator related to the aplastic anemia; an additional karyotype sample collected from this patient 4 months later did not reveal any abnormalities and at Week 26 this karyotype returned to normal. The abnormal karyotype reported at Week 13 was 46,XY,i(17)(q10)[5]/45,idem,-21[6]/46,XY[4]. This karyotype return to normal at Week 26: 46,XY[20]. No patient had clonal evolution to paroxysmal nocturnal hemoglobinuria.

Serial intensive pharmacokinetic blood samples were collected from 12 patients for the initial starting dose of 25 mg of eltrombopag daily (Supplementary Tables S4 and S5) and the individual concentration–time profiles by linear and semi-logarithmic scale are presented (Supplementary Fig. S5). After 2 weeks of eltrombopag administration at 25 mg/d, the median time to reach maximum plasma concentration was 3.73 h. The geometric mean ratio (Geo-mean) and geometric coefficient of variation (Geo-CV%) of the maximum plasma concentration was 2,960 ng/mL (67.0%), and the Geo-mean (and Geo-CV%) of the area under the plasma concentration–time curve from zero (pre-dose) to the last quantifiable sample time was 53,900 h*ng/mL (67.6%). The Geo-mean (Geo-CV%) apparent systemic clearance of the drug at steady state from the plasma was 0.405 L/h (47.2%). With increasing eltrombopag dose from 25 to 150 mg/d, the Geo-mean plasma trough concentrations increased from 1,780 to 19,900 ng/mL, with Geo-CV% variability ranging from 57.8–75.2% (Supplementary Table S5). The observed greater than dose-proportional increase in plasma through eltrombopag concentrations between 25 and 75 mg is consistent with historical eltrombopag pharmacokinetics performance, as is the generally dose-proportional increase between 75 and 150 mg.

## Discussion

In this study, we evaluated the efficacy and safety of eltrombopag in Chinese patients with r/r SAA. Overall, 70% (14/20) of patients met the primary endpoint of HR rate at Week 26, with 64% (9/14) of these responders having at least a bi-lineage response. The HR at Week 13 was met in 65% (13/20) of patients, and the clinical benefit of reducing the transfusion need was also observed in both studied time points, suggesting that patients receiving eltrombopag experience an early response. The reduction in transfusion need is associated with clinical benefits such as reduced mortality and length of hospitalization, as well as reduced management costs. AEs reported were generally mild, with no treatment-related deaths reported. There were no new safety signals identified throughout this study. A major complication associated with long-term treatment of SAA is clonal evolution, which globally occurs in up to 15% of patients after IST^14^. Here, we report one case of transient clonal evolution, which was deemed to be related to aplastic anemia by the investigator. The key limitations of this study are its small sample size and the trial design, which did not involve masking of the treatment arm, nor the inclusion of a control arm. However, this study demonstrates the feasibility of eltrombopag treatment in Chinese patients with r/r SAA and warrants further studies with a higher number of Chinese patients.

The reported results in this study show a similar improvement trend as in previously published data, where the treatment with eltrombopag was associated with multilineage clinical response in patients with r/r SAA^6, 7^. In fact, results from an interventional, nonrandomized phase 2 clinical trial based in the United States, investigating the efficacy of eltrombopag in 25 patients with r/r SAA showed that 44% (11/25) of patients enrolled into the trial had at least uni-lineage response to eltrombopag treatment at 12 weeks^7^. In the follow-up to this US study, the overall response rate at 3 to 4 months was 40% (17/43), which included bi-lineage and tri-lineage responses, and the 12-month extension phase continued to show improvements in response^6^. There is limited evidence supporting the use of eltrombopag in Chinese patients with SAA, which involves a combination regimen only and has small patient numbers^15^. However, eltrombopag has previously been studied in an East Asian patient population for the treatment of immune thrombocytopenia, where a lower initial dose was used because of higher plasma exposure in this patient population^16^. Eltrombopag was shown to be efficacious in elevating platelet counts and the reduction of bleeding events, with a safety profile comparable to the well-established safety profile of eltrombopag globally^16^.

In summary, although this study is limited by the small patient population, it is the first prospective study to demonstrate the safety and efficacy of treatment with eltrombopag in a Chinese adult patient population with r/r SAA. These findings are promising and highlight the potential therapeutic benefit of eltrombopag in this specific population with high unmet need. 

### Supplementary Information

Supplementary Information.

## Data Availability

Novartis is committed to sharing with qualified external researchers access to patient-level data and supporting clinical documents from eligible studies. These requests are reviewed and approved by an independent review panel on the basis of scientific merit. All data provided are anonymized to respect the privacy of patients who have participated in the trial, in line with applicable laws and regulations. This trial data availability is in accordance with the criteria and process described on http://www.clinicalstudydatarequest.com.
