# Supplementary material for: Efficacy and safety of eltrombopag in Chinese patients with refractory or relapsed severe aplastic anemia
Source: Sci Rep. 2023 Nov 2;13:18955. doi: 10.1038/s41598-023-45607-0 (PMC10622422; doi:10.1038/s41598-023-45607-0)
Supplement: Supplementary file 1 — Supplementary Information. [file 41598_2023_45607_MOESM1_ESM.docx]

**Supplementary Information**

Efficacy and safety of Eltrombopag in Chinese patients with refractory or relapsed severe aplastic anemia

Hong Chang^1^, Guangsheng He^2^, Rong Fu^3^, Fei Li^4^, Bing Han^5^, Tao Li^6^, Lei Liu^6^, Hemant Mittal^7^, Hantao Jin^6^, Fengkui Zhang^8^

^1^West China Hospital of Sichuan University, Chengdu, China

^2^The First Affiliated Hospital of Nanjing Medical University, Jiangsu Province Hospital, Collaborative Innovation Center for Cancer Personalized Medicine, Nanjing, China

^3^Tianjin Medical University General Hospital, Tianjin, China

^4^The First Affiliated Hospital of Nanchang University, Nanchang, China

^5^Peking Union Medical College Hospital, Beijing, China

^6^Novartis Pharma Co., Ltd.; Beijing, China

^7^Novartis Healthcare Private Limited, Hyderabad, India

^8^Institute of Hematology & Blood Disease Hospital, Chinese Academy of Medical Sciences & Peking Union Medical College, Tianjin, China

**Corresponding author:**

Fengkui Zhang

Institute of Hematology & Blood Disease Hospital, Chinese Academy of Medical Sciences & Peking Union Medical College

Phone no: +86 13821700281

Email: [fkzhang@ihcams.ac.cn](mailto:fkzhang@ihcams.ac.cn)

**Supplementary Fig.S1** Study design^a^


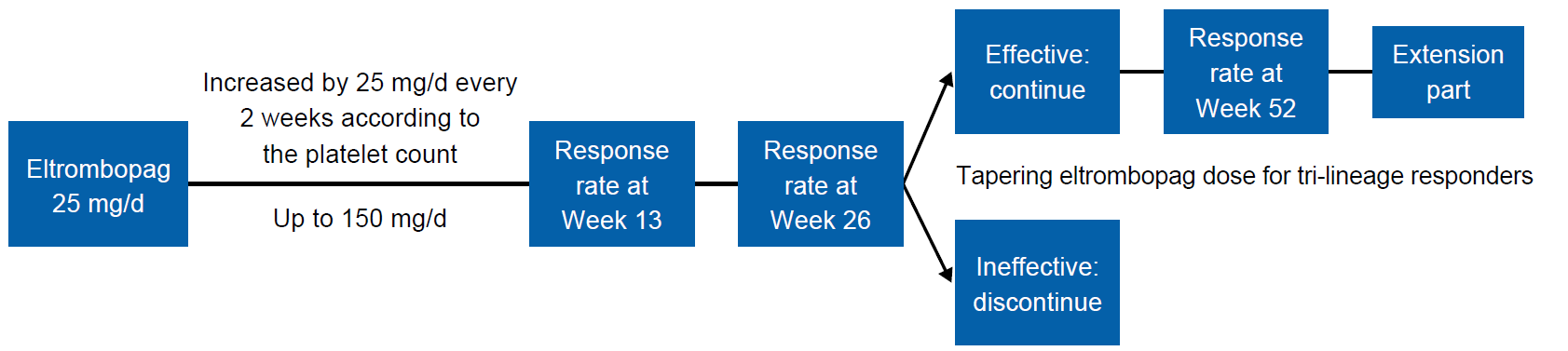


^a^This manuscript only reports on the data available up to the cutoff date (July 16, 2021)

**Supplementary Fig.S2** Patient disposition^a^ (full analysis set)


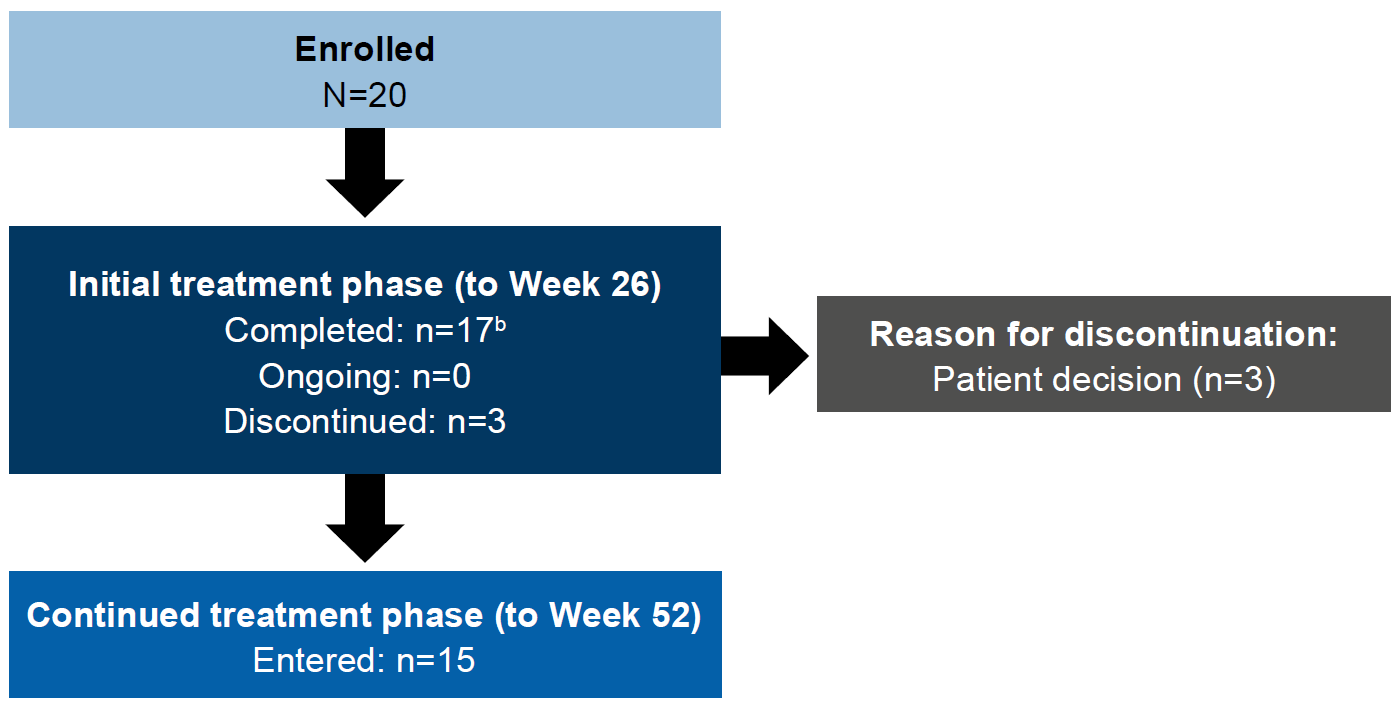


^a^A total of 24 patients were screened for this study, with 4 patients classified as screening failures

^b^Two patients completed the 26-week treatment phase but were considered as non-responders by the investigator at Week 26. Eltrombopag was discontinued in these patients in accordance with the study protocol

**Supplementary Fig. S3** Supplementary analysis of the derived hematologic response rate, according to IWG criteria (**a**) and number of responders with uni-, bi-, or tri-lineage response (**b**) at Week 26 (full analysis set)^a^


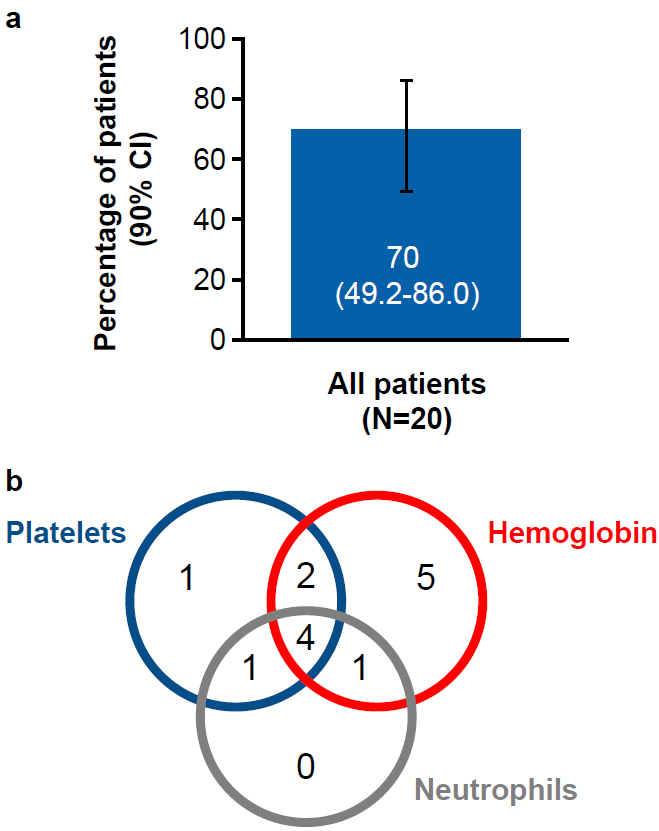


^a^In this supplementary analysis, the hematologic response rate was derived programmatically using laboratory results and transfusion records instead of the hematologic response reported by the investigators in the response assessment case report form

CI, confidence interval; IWG, International Working Group

**Supplementary Fig.S4** Plots of median (IQR) platelet counts (**a**) hemoglobin levels (**b**) and neutrophil counts over time (**c**) (full analysis set)


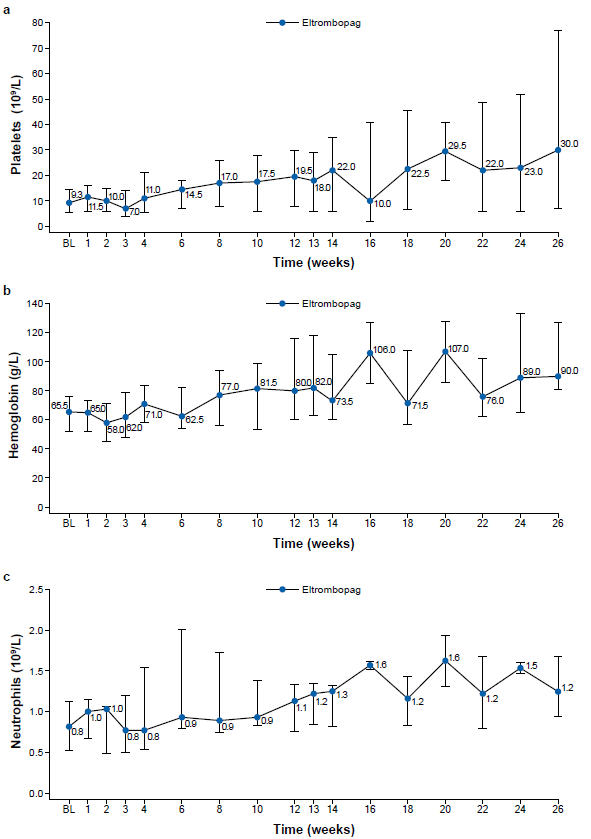


The dots in the plots represent the median values. Whiskers (vertical lines) extend to the IQR range (short horizontal lines). Values outside this range are not displayed.

Data beyond Week 26 are not displayed

IQR, interquartile range

**Supplementary Fig. S5** Linear (**a**) and semi-logarithmic (**b**) view of concentration-time profile with serial intensive samples at steady state of eltrombopag 25 mg/d (n=12) (pharmacokinetic analysis set)


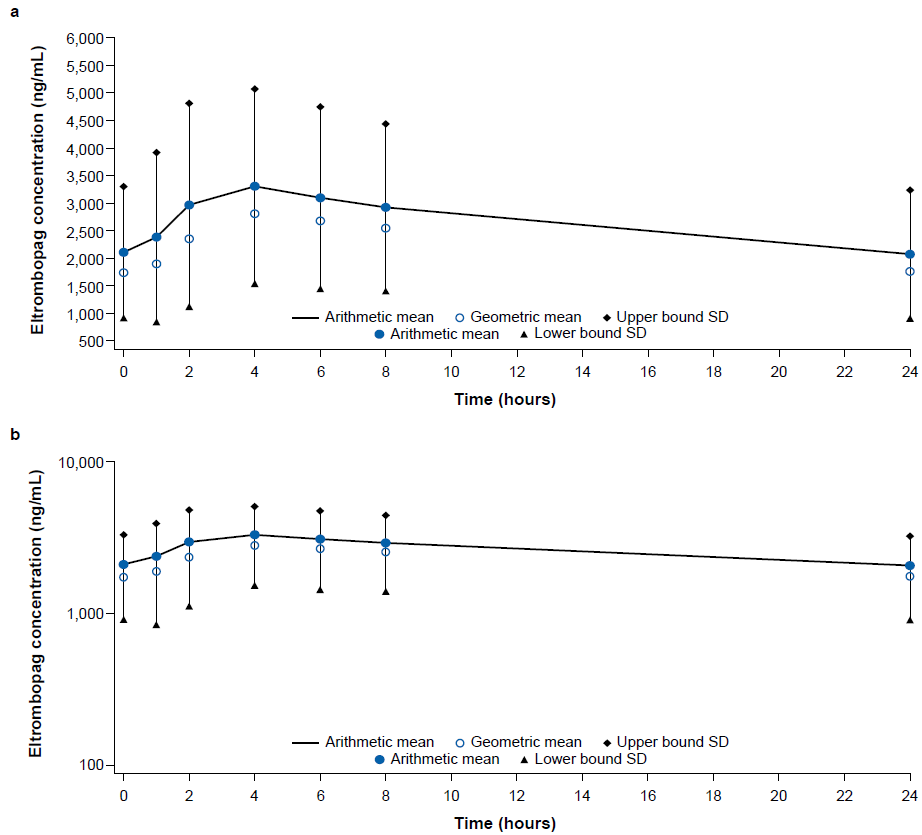


Zero concentrations at individual timepoints are excluded from geometric mean computation

SD, standard deviation

**Supplementary Table S1** IWG criteria [1] for hematologic response rate

| **Assessment item** | **Baseline transfusion status** | **Response criteria** |
| --- | --- | --- |
| Platelet count | Platelet transfusion independent | Transfusion independent and increase in platelet count of ≥20×10^9^/L or more from baseline |
|  | Platelet transfusion dependent | No platelet transfusion requirement for 8 weeks |
| Hemoglobin level | RBC transfusion independent | When the baseline hemoglobin level is <90×10^9^/L: transfusion independent and increase in platelet count of ≥15×10^9^/L from baseline |
|  | RBC transfusion dependent | A decrease of ≥4 units^a^ in RBC transfusions in the post-treatment 8-week period  Or no RBC transfusion requirement for 8 weeks (<4 units^a^ RBC in 8-week period at baseline) |
| Neutrophil count | NA | In the absence of G-CSF taken within 21 days preceding the blood sample collection, increase from baseline by ≥0.5×10^9^/L, or (if <0.5×10^9^/L at baseline) increase by ≥100% |

^a^1 unit – RBC derived from 200 mL of blood

G-CSF, granulocyte colony stimulating factor; IWG, International Working Group; NA, not applicable; RBC, red blood cell

**Supplementary Table S2** Most common adverse events (reported in ≥10% of patients) by preferred term (safety set)

|  | **Eltrombopag**  **N=20** |  |
| --- | --- | --- |
| **Preferred term** | **All grades**  **n (%)** | **Grade ≥3**  **n (%)** |
| **Number of patients with ≥1 event** | **19 (95.0)** | **7 (35.0)** |
| Upper respiratory tract infection | 6 (30.0) | 2 (10.0) |
| Blood creatinine increased | 5 (25.0) | 1 (5.0) |
| Hyperuricemia | 5 (25.0) | 0 |
| Hyperbilirubinemia | 4 (20.0) | 0 |
| Pyrexia | 4 (20.0) | 1 (5.0) |
| Blood glucose increased | 3 (15.0) | 0 |
| Hypertriglyceridemia | 3 (15.0) | 1 (5.0) |
| Edema peripheral | 3 (15.0) | 0 |
| Alanine aminotransferase increased | 2 (10.0) | 0 |
| Cataract | 2 (10.0) | 0 |
| Cough | 2 (10.0) | 0 |
| Diarrhea | 2 (10.0) | 0 |
| Drug-induced liver injury | 2 (10.0) | 0 |
| Epistaxis | 2 (10.0) | 1 (5.0) |
| Hepatic function abnormal | 2 (10.0) | 0 |
| Hypercholesterolemia | 2 (10.0) | 1 (5.0) |
| Hyperlipidemia | 2 (10.0) | 0 |
| Hyponatremia | 2 (10.0) | 1 (5.0) |
| Hypoproteinemia | 2 (10.0) | 1 (5.0) |
| Intentional product misuse | 2 (10.0) | 0 |
| Iron overload | 2 (10.0) | 0 |
| Liver injury | 2 (10.0) | 0 |
| Mouth ulceration | 2 (10.0) | 0 |
| Pneumonia | 2 (10.0) | 2 (10.0) |

Numbers (n) represent counts of patients

A patient with multiple severity grades for a preferred term is only counted under the maximum grade

MedDRA version 24.0, CTCAE version 4.03

AE, adverse event; CTCAE, Common Terminology Criteria for Adverse Events; MedDRA, Medical Dictionary for Regulatory Activities

**Supplementary Table S3** Adverse events of special interest ─ hepatotoxicity and acute kidney injury measures (safety set)

| **Hepatotoxicity (all indications)** | **Eltrombopag**  **N=20**  **n (%)** |
| --- | --- |
| **Number of patients with ≥1 event** | **10 (50.0)** |
| Drug induced liver injury | 2 (10.0) |
| Hepatic function abnormal | 2 (10.0) |
| Hyperbilirubinemia | 4 (20.0) |
| Liver injury | 2 (10.0) |
| Alanine aminotransferase increased | 2 (10.0) |
| Aspartate aminotransferase increased | 1 (5.0) |
| Blood alkaline phosphatase increased | 1 (5.0) |
| Blood bilirubin increased | 1 (5.0) |
| **Treatment-related AEs** | **9 (45.0)** |
| Dose not changed/NA/Unknown | 10 (50.0) |
| Medication or therapy taken | 4 (20.0) |
| **AE outcome** |  |
| Recovered/resolved | 7 (35.0) |
| Not recovered/not resolved | 5 (25.0) |
| **Acute Kidney Injury** | **n (%)** |
| **Number of patients with ≥1 event** | **6 (30.0)** |
| Blood creatinine increased | 5 (25.0) |
| Blood urea increased | 1 (5.0) |
| Glomerular filtration rate decreased | 1 (5.0) |
| Renal impairment | 1 (5.0) |
| **Maximum grade** |  |
| Grade 3 AEs | 1 (5.0) |
| **Treatment-related AEs** | 3 (15.0) |
| Dose not changed/NA/Unknown | 6 (30.0) |
| **AE outcome** |  |
| Recovered/resolved | 3 (15.0) |
| Not recovered/not resolved | 4 (20.0) |

Numbers (n) represent counts of patients. A patient may be counted in several rows for action taken and outcome

MedDRA version 24.0, CTCAE version 4.03

AE, adverse event; CTCAE, Common Terminology Criteria for Adverse Events; MedDRA, Medical Dictionary for Regulatory Activities; NA, not applicable

**Supplementary Table S4** Summary of plasma pharmacokinetic parameters of eltrombopag (pharmacokinetic population)

|  | **Eltrombopag (N=20)**  **Dose=25 mg** | | | | |
| --- | --- | --- | --- | --- | --- |
| **Dose Statistics** | **C_max_**  **(ng/mL)**  **n=12** | **T_max_**  **(h)**  **n=12** | **AUC_tau_**  **(h*ng/mL)**  **n=11** | **AUC_last_**  **(h*ng/mL)**  **n=12** | **CL_ss_/F**  **(L/h)**  **n=11** |
| Mean (SD) | 3,450 (1,900) |  | 67,900 (31,400) | 62,700 (33,200) | 0.441 (0.178) |
| CV% | 55.2 |  | 46.3 | 53.0 | 40.4 |
| Geo-mean | 2,960 |  | 61,800 | 53,900 | 0.405 |
| Geo-CV% | 67.0 |  | 47.2 | 67.6 | 47.2 |
| Median | 2,520 | 3.73 | 51,200 | 50,700 | 0.488 |
| Min; max | 756; 7,490 | 0.917; 5.90 | 36,900; 124,000 | 13,100; 123,000 | 0.202; 0.677 |

N: Number of patients with corresponding evaluable PK parameters

CV% = SD/mean*100; Geo-CV% = sqrt (exp [variance for log transformed data]-1)*100

AUC_last_, area under the plasma concentration-time curve from zero (pre-dose) to the last quantifiable sample time; AUC_tau_, area under the plasma concentration-time curve calculated to the end of a dosing interval (tau) at steady-state; CL_ss_/F, apparent systemic (or total body) clearance at steady state from plasma; C_max_, maximum (peak) observed plasma drug concentration after single dose of administration; Geo-CV, geometric coefficient of variation; Geo-mean, geometric mean ratio; PK, pharmacokinetic; SD, standard deviation; T_max_, time to reach peak plasma drug concentration

**Supplementary Table S5** Summary of plasma trough concentration (ng/mL) of eltrombopag (pharmacokinetic population)

|  | **Eltrombopag (N=20)** | | | | |  | |
| --- | --- | --- | --- | --- | --- | --- | --- |
| **Dose Statistics** | **25 mg**  **n=20** | **50 mg**  **n=16** | **75 mg**  **n=19** | **100 mg**  **n=19** | **125 mg**  **n=15** | | **150 mg n=15** |
| Mean (SD) | 2,180  (1,490) | 5,180 (3,320) | 11,300 (6,000) | 14,700 (8,070) | 20,300 (11,300) | | 23,800 (13,700) |
| CV% | 68.7 | 64.2 | 53.2 | 54.9 | 55.7 | | 57.7 |
| Geo-mean | 1,780 | 4,270 | 9,870 | 12,300 | 17,500 | | 19,900 |
| Geo-CV% | 72.5 | 75.2 | 57.8 | 75.1 | 64.6 | | 74.1 |
| Median | 1,670 | 4,050 | 9,290 | 13,500 | 16,900 | | 20,800 |
| Min; max | 452;  6,620 | 916; 13,600 | 3,770; 24,200 | 2,380; 34,100 | 5,310; 43,800 | | 4,390; 46,700 |

N: Number of patients with corresponding evaluable PK parameters

CV% = SD/mean*100; Geo-CV% = sqrt (exp [variance for log transformed data]-1)*100

Geo-CV, geometric coefficient of variation; Geo-mean, geometric mean ratio; PK, pharmacokinetic; SD, standard deviation

**Supplementary references**

1. Pulsipher MA, Young NS, Tolar J, Risitano AM, Deeg HJ, Anderlini P, Calado R, Kojima S, Eapen M, Harris R, Scheinberg P, Savage S, Maciejewski JP, Tiu RV, DiFronzo N, Horowitz MM, Antin JH. Optimization of therapy for severe aplastic anemia based on clinical, biologic, and treatment response parameters: conclusions of an international working group on severe aplastic anemia convened by the Blood and Marrow Transplant Clinical Trials Network, March 2010. Biol Blood Marrow Transplant. 2011;17(3):291-299. <https://doi.org/10.1016/j.bbmt.2010.10.028>
